# Advanced Pore Structure Characterization of High-Volume Mineral Admixture Steam-Cured Mortar Using X-Ray Computed Tomography

**DOI:** 10.3390/ma18071575

**Published:** 2025-03-31

**Authors:** Yuntian Wang, Songlin Xie, Yushu Li, Min Yang, Qiuling Chen, Lijuan Huang, Danping Hu, Sheng Li

**Affiliations:** 1 School of Urban Construction, Chengdu Polytechnic, Chengdu 610041, China; wangyuntian@cdp.edu.cn (Y.W.); scatcliyushu@gmail.com (Y.L.); yangmin1975@foxmail.com (M.Y.); cql0709@126.com (Q.C.); 17835111665@163.com (L.H.); 2 Sichuan Thermoelectric Materials and Devices Application Engineering Research Center, Chengdu 610041, China; 3 Luzhou Key Laboratory of Intelligent Construction and Low-Carbon Technology, Luzhou 646000, China; hdpshq@163.com; 4 Central & Southern China Municipal Engineering Design & Research Institute Co., Ltd., Wuhan 430010, China; xiesl920518@163.com; 5 LVTC School of Intelligent Construction, Luzhou Vocational and Technical College, Luzhou 646000, China; 6 College of Civil Engineering, Lanzhou Jiaotong University, Lanzhou 730070, China

**Keywords:** steam curing, pore structure, X-ray CT, sphericity, fractal dimension, cementitious materials

## Abstract

Steam curing is a widely used method in the production of industrial precast concrete but it often leads to thermal damage that negatively impacts the material’s long-term durability and mechanical strength. The use of supplementary cementitious materials (SCMs) has shown considerable promise in improving pore structure and alleviating these adverse effects. This study employs high-resolution X-ray computed tomography (X-CT) to thoroughly assess how steam curing temperatures and various subsequent curing regimes influence the pore characteristics of mortars containing high volumes of mineral admixtures. The results shows that steam-cured specimens under water curing (ST8012-WA) achieved a compressive strength of 51.72 MPa and flexural strength of 5.85 MPa, representing improvements of 9% and 19.8%, respectively, compared to natural curing (ST8012-NA: 47.32 MPa and 4.88 MPa). The standard-cured specimen (SD) exhibited the highest compressive strength of 54.18 MPa, highlighting the detrimental effects of elevated steam curing temperatures. The findings reveal that higher steam curing temperatures result in increased porosity and decreased mechanical strength, challenges that can be effectively mitigated through appropriate postcuring techniques. Notably, water curing following steam curing proves especially effective in reducing pore size variability and improving the material’s durability. This research offers new insights into the intricate relationships among curing temperature, pore morphology, and mechanical performance, providing practical recommendations to optimize the quality and longevity of steam-cured precast concrete components.

## 1. Introduction

Concrete is the most extensively utilized construction material worldwide, with its production quality playing a pivotal role in driving infrastructure development [1,2]. Among the factors influencing concrete quality, the curing process is one of the most critical, as it directly affects the microstructural development and long-term durability of the material [3]. Steam curing is a prevalent technique employed in industrial precast concrete production. It facilitates the rapid hydration of cement, particularly within the initial 24 h, by creating a high-temperature, high-humidity environment. This process enhances early-age strength and productivity by accelerating microstructural formation [4,5,6]. However, the elevated temperatures associated with steam curing often induce microstructural changes such as increased porosity and reduced mechanical strength, which negatively impact the material’s long-term durability.

Recent advancements in steam curing have aimed to balance early strength development with enhanced long-term performance. However, studies highlight trade-offs associated with steam curing, including stagnation in strength progression and a decrease in resistance to freeze-thaw cycles [7,8,9,10]. To address these challenges, high-volume mineral admixtures such as fly ash and slag have gained attention for their ability to refine pore structures and mitigate thermal damage. Through pozzolanic reactions, these materials improve microstructural integrity by reducing the heat of hydration and limiting the formation of harmful pore networks. However, despite these developments, critical gaps remain in understanding how variations in steam curing temperatures influence pore morphology evolution and how these microstructural changes relate to mechanical performance over time.

Numerical modeling effectively resolves multiphysics coupling in steam curing. The transient activation energy algorithm [11] predicts hydration heat dynamics under atmospheric steam curing, showing prolonged precuring (>7 h) reduces peak heat rates by 12–18%. DEM (discrete element method) simulations [12] with steam curing damage indices demonstrate recycled concrete’s triaxial degradation: temperatures exceeding 40 °C exacerbate internal damage and alter failure modes to compression-dominated. These models quantify temperature-dependent thermomechanical behavior, supporting the optimization of steam curing regimes.

The majority of the existing experimental literature focuses on the macroscopic properties of steam-cured concrete, such as compressive and flexural strength, while providing limited insight into the detailed characterization of pore structures. Advanced nondestructive techniques have been underutilized in this area [13,14]. Although widely applied, traditional methods such as mercury intrusion porosimetry (MIP) and scanning electron microscopy (SEM) have notable limitations. MIP often alters the pore structure during sample preparation and SEM cannot provide three-dimensional visualizations of small pores. These constraints impede a holistic understanding of pore characteristics and their impact on concrete properties [15]. To address these limitations, this study employs high-resolution X-ray computed tomography (X-CT), an advanced nondestructive imaging technique capable of three-dimensional analysis of pore structures at various scales. X-CT has demonstrated remarkable effectiveness in visualizing and quantifying multiscale pores in a range of materials, including coal seams, porous metals, gas hydrates, and permeable concrete [16,17]. Moreover, different studies utilize advanced imaging techniques such as X-CT and 3D volume analysis to investigate the microstructural properties of various concrete types, including recycled, high-performance, and phase-change materials [18,19,20,21]. However, the application of X-CT for the pore morphology of mortar requires further attention owing to the scarcity of the literature and practical demand of determining the pore morphology of mortar for various applications. The current study leverages X-CT to provide a detailed and accurate assessment of the evolution of pore morphology in high-volume mineral admixture mortars subjected to varying steam curing temperatures and postcuring regimes.

The novelty of this research lies in its systematic application of X-CT to investigate the effects of steam curing on the pore characteristics of mortars containing high volumes of mineral admixtures. Unlike prior studies, this work emphasizes the interaction between curing parameters and microstructural properties, offering a three-dimensional perspective on pore morphology. By examining the impact of different steam curing temperatures and subsequent curing methods, this study provides critical insights into optimizing curing processes to enhance both early-age strength and long-term durability. The findings contribute to bridging the gap between microstructural properties and macroscale performance, offering practical strategies to mitigate the adverse effects of high-temperature curing in industrial applications. These insights pave the way for more durable, efficient, and sustainable precast concrete production methods, addressing the evolving demands of modern infrastructure.

## 2. Methods

### 2.1. Materials

The mortar used in this study was prepared using C50 grade cement, specifically P.O 42.5 Hailuo Ordinary Portland cement, supplemented with S95 granulated blast-furnace slag and Class II fly ash. It is important to note that the P.O 42.5 cement contains 6–15% mineral additions during the grinding process, as specified by GB175. As such, it is essential to differentiate the effects of these inherent mineral additions from those of the supplementary fly ash and slag, as they all contribute to the overall impact on the pore structure. The chemical composition of the cement, slag, and fly ash used in this study is outlined in Table 1, offering insight into how these materials influence hydration and the development of the pore structure. Natural quartz sand is used as a fine aggregate with a particle size range of 0.1–2 mm, conforming to the Chinese standard GB/T 14684-2022 [22]. The mixing water was tap water with a pH of 7.2 and total dissolved solids (TDS) of 150 mg/L.

### 2.2. Specimen Preparation and Curing Regimes

Mortar specimens were prepared following the guidelines outlined in GB/T 17671-2021 [23], cast into steel molds with dimensions of 40 mm × 40 mm × 160 mm, and compacted using vibration. The specimens underwent various curing regimes to investigate the effects of initial steam curing at elevated temperatures, followed by different postcuring treatments on their long-term properties. The curing procedures included standard curing (SD) as well as steam curing at 40 °C, 60 °C, and 80 °C, each followed by one of three posttreatment options: natural curing (NA), water curing (WA), or SD. Each curing regime was applied to three specimens to ensure statistical reliability. An optimized steam curing protocol, determined through preliminary testing, consisted of four stages: holding at 20 °C for 3 h, heating for 2 h, maintaining at 80 °C for 12 h, and cooling for 2 h. After steam curing, the specimens were demolded within 1 h and categorized into three groups: natural curing (ST-NA), standard curing (ST-SD), and water curing (ST-WA). X-ray computed tomography (X-CT) was employed to assess the pore structure of the specimens after 28 days of curing, allowing for a comprehensive evaluation of the effects of initial steam curing at high temperatures and subsequent curing methods on the pore structure and durability of the mortar. The curing scheme and scanning pattern of different mortar specimens are detailed in Table 2.

### 2.3. Experimental Setup and Testing

The experimental setup involved continuous monitoring of the temperature profiles during the steam curing process to ensure consistency across all specimens. The steam curing procedure entailed a gradual increase in temperature to the target values, followed by a prolonged period at these temperatures to facilitate optimal hydration. After the steam curing phase, a series of postcuring treatments were applied to evaluate their effectiveness in reducing thermal damage. Then, 28-day mechanical property tests were conducted on the specimens using a Wance microcomputer-controlled electronic universal testing machine in accordance with GB/T 17671-2021 standard. Additionally, the mass loss of the specimens during steam curing was recorded to assess the impact of elevated temperatures on water retention and the efficiency of hydration.

### 2.4. CT Imaging

#### 2.4.1. CT Imaging Principle

Computed tomography (CT) is a three-dimensional imaging modality that reconstructs the external and internal features of an object into a volumetric 3D representation by integrating a series of projected two-dimensional (2D) images. In this technique, X-rays are directed at the specimen from multiple angles and the attenuation of the X-ray beams is governed by the specimen’s density, thickness, and pore structure. The transmitted photons, which encode information regarding this attenuation, are captured by a detector comprising photocells. The detector converts the X-ray energy into visible light, which is subsequently processed into 2D grayscale images. Through computational processing and the stacking of a substantial number of these grayscale images, a high-resolution 3D reconstruction of the specimen is achieved [24,25,26].

#### 2.4.2. CT Image Processing Method

This study conducted CT imaging using the German Vtomexs microfocus X-ray CT system. The Vtomexs system is equipped with a maximum tube power of 320 W, a voltage range for the X-ray source of 10–225 kV, and a tube current range of 0.01–3.0 mA. Mortar samples, prepared as 20 mm cubes, were subjected to X-CT scanning after 28 days of curing.

The cement paste samples, with a diameter of 14 mm and a height of 20 mm, were mounted on a carrier table positioned between the X-ray source and the plate detector using a sample holder. The carrier table was able to rotate around a fixed axis, ensuring proper alignment between the X-ray source, the sample, and the detector. The distances from the sample to the X-ray source and detector were 47.5 mm and 260 mm, respectively. The scanning voltage was set to 95 kV, with a tube current of 105 μA. The scanning procedure involved a full 360° rotation with an angular step size of 0.24°, resulting in 1501 projections, each with an exposure time of 7.0 s. A flat-panel detector with a resolution of 2000 × 2000 pixels was used. The total scanning time for each specimen was approximately 4 h, which included the acquisition of reference images and the reconstruction of projections. Under these conditions, a stack of 1012 × 1024 2D images was generated, with a pixel size of around 10 μm.

CT images often display artifacts caused by beam hardening, which typically appear as darker centers and brighter edges. Using a fixed grayscale threshold to segment pores may result in inaccuracies such as missing pores in the brighter areas or overestimating pore sizes in the darker regions. To improve the accuracy of pore segmentation, we applied an active contour model during the image preprocessing stage. This technique involves creating a deformable initial contour around the target region and defining an energy functional equation. The contour, influenced by both its intrinsic deformation energy and the external potential energy near the boundary, adjusts to the target image’s edge by minimizing the functional energy. This approach ensures the identification of the optimal boundary contour. The energy function at the control points of the initial contour is defined as [27]:

(1) $$E_{tot}=\int_{0}^{1}\left.\left[E_{int}\left(v\left(s\right)\right)+E_{ext}\left(v\left(s\right)\right)\right.\right]ds$$

where $E_{int}$ denotes the internal energy, governing the elastic deformation of the active contour curve; $E_{ext}$ represents the external potential energy, attracting the active contour curve to the target feature region in the image; *s* is the normalized arc length; and $v\left(s\right)=\left(x\left(s\right),\;y\left(s\right)\right)$ denotes the two-dimensional coordinates of the control points on the active contour curve.

In practical computations, the discrete equation of the energy function is often used to calculate $E_{tot}$.

(2) $$E_{tot}=\sum_{\left\{i=1\right\}}^{N}\left.\left[\alpha {\left(v_{i}\right)\left(v_{i+1}-v_{i}\right)}^{2}+\beta {\left(v_{i}\right)\left(v_{i+1}-2v_{i}+v_{i-1}\right)}^{2}+E_{ext}\left(v_{i}\right)\right.\right]$$

where, $v_{i}$ represents the *i* discrete control point on the active contour curve; $v_{i}-v_{i-1}$ is the first-order derivative and $v_{i+1}-2v_{i}+v_{i-1}$ is the second-order derivative of the control points on the active contour curve; *n* is the number of control points; *α* is the elasticity coefficient; and *β* is the stiffness coefficient.

The minimization of the energy function is achieved by employing the Euler equation:

(3) $$\alpha v^{\prime \prime }-\beta v^{4}\left(s\right)-\nabla E_{ext}=0$$

where $v^{\prime \prime }\left(s\right)$ and $v^{4}\left(s\right),$ respectively, denote the second- and fourth-order derivatives of the control points in the image space.

**Figure 1:**
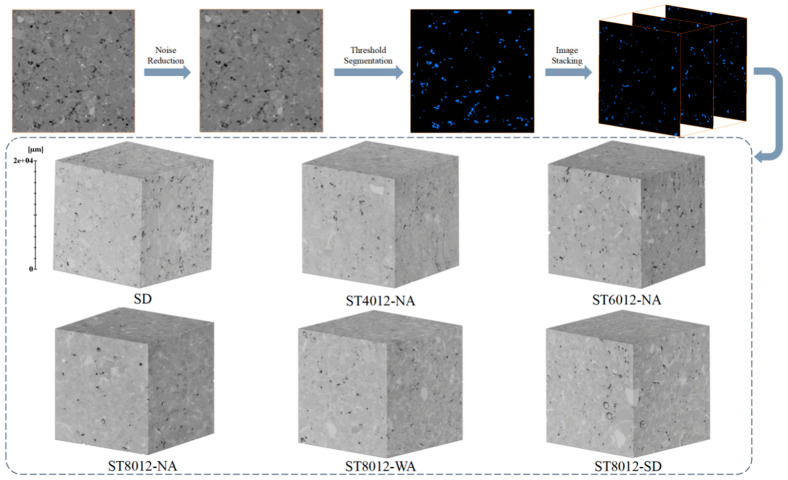
CT image analysis and reconstruction process.

At this stage, the image segmentation problem is reformulated as a variational problem. Under the discrete conditions of grayscale digital images, a system of linear equations is established and solved iteratively. After segmenting the pores using the active contour model, a three-dimensional reconstruction of the pore structure is carried out. Subsequently, a statistical analysis of the pore structure characteristics is performed using Python (https://www.python.org/). The CT image processing procedure described above is illustrated in Figure 1.

## 3. Results and Discussion

### 3.1. Pore Size Distribution Analysis

**Figure 2:**
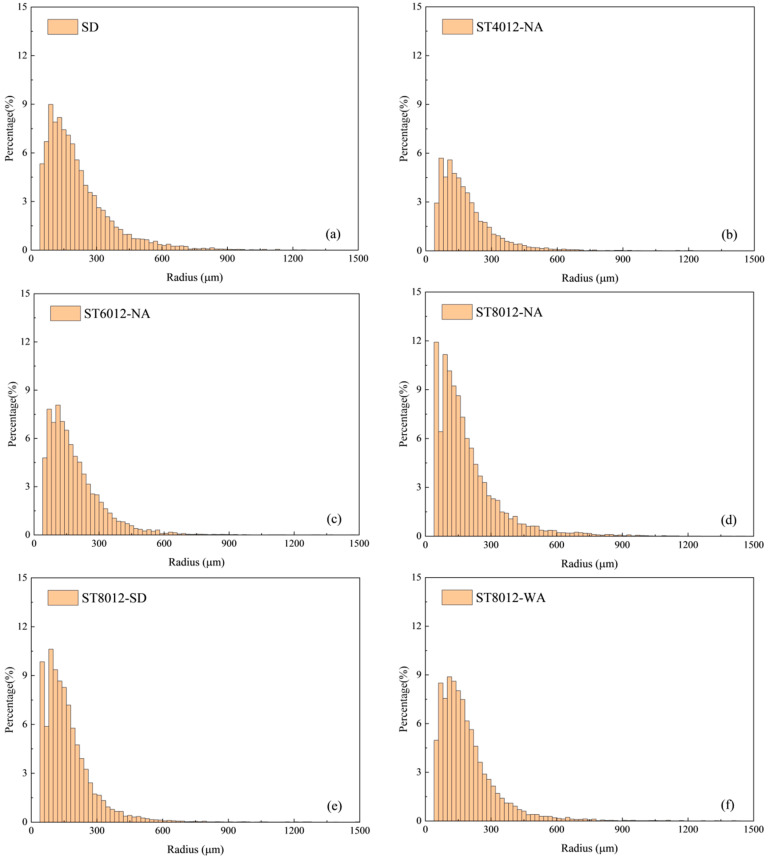
Pore size distribution analysis of cured mortar specimens. (**a**) Pore size distribution of SD sample. (**b**) Pore size distribution of ST4012-NA sample. (**c**) Pore size distribution of ST6012-NA sample. (**d**) Pore size distribution of ST8012-NA sample. (**e**) Pore size distribution of ST8012-SD sample. (**f**) Pore size distribution of ST8012-WA sample.

The reconstructed three-dimensional models of the mortar specimens revealed a wide range of pore sizes distributed across the surface. A comprehensive quantitative analysis of the pore size distribution was conducted under six different curing conditions and the statistical results are presented in Figure 2. This analysis highlights how various curing treatments affect the pore characteristics within the specimens.

The results from the 3D models show that steam curing, particularly at higher temperatures, significantly increases the proportion of larger pores (ranging from 0–300 μm), with the most pronounced effect observed at 80 °C. This increase in pore size is directly linked to a rise in overall porosity, which, in turn, leads to a notable reduction in the mechanical strength of the mortar. These findings underscore the critical influence of steam curing conditions, particularly temperature, on the pore structure and structural integrity of the material. Specifically, as the curing temperature increases, the number of large pores also increases, illustrating the sensitivity of pore formation to thermal conditions during curing. Additionally, specimens subjected to water curing following steam curing exhibited a notable reduction in pore size heterogeneity. This suggests that appropriate postcuring, especially water curing, can effectively mitigate some of the adverse effects of high-temperature steam curing. These results highlight the practical significance of postcuring in enhancing the long-term durability of concrete. In real-world construction applications, ensuring that steam-cured components undergo subsequent water curing could be an essential strategy to reduce pore heterogeneity and improve the material’s long-term strength, thereby contributing to more resilient and durable infrastructure.

The impact of supplementary cementitious materials (SCMs) on pore size distribution was also evident in the study. Specimens with higher SCM content showed a lower proportion of large pores compared to those without SCMs, highlighting the potential of these materials to refine the pore structure. This finding is crucial as it demonstrates that SCMs can alleviate some of the negative effects induced by high-temperature steam curing. By incorporating SCMs into the mix design for steam-cured concrete, it is possible to optimize the pore characteristics, which ultimately enhances the durability and mechanical properties of the material. The adoption of SCMs in field applications, particularly in precast concrete production, could be highly beneficial. This approach not only ensures rapid strength development but also guarantees improved long-term durability, addressing the need for both early-age performance and sustained resilience in infrastructure projects. These findings suggest several practical implications for the construction industry. First, controlling the steam curing temperature is essential to prevent excessive porosity and maintain the structural integrity of concrete components. Second, incorporating postcuring techniques, such as water curing, can significantly enhance the durability and long-term strength of steam-cured concrete. Finally, the integration of SCMs into the concrete mix design presents a promising strategy to optimize pore structure and improve the overall performance of concrete, particularly in precast applications where both rapid strength gain and long-term durability are critical. By applying these strategies, engineers and construction professionals can develop more sustainable, resilient, and high-performance concrete materials for infrastructure development.

### 3.2. Fractal Analysis

The application of fractal dimension and fractal geometry provides a robust method for quantitatively characterizing irregular geometries and spatial heterogeneity. Several studies have highlighted the efficacy of fractal theory in describing the complexity of pore structures, both in two-dimensional and three-dimensional spaces [28]. In this study, the fractal dimension of steam-cured mortar was determined using the box-counting method [29].

**Figure 3:**
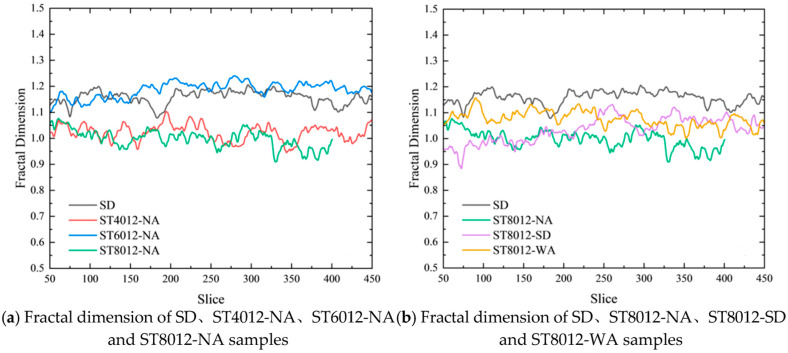
Fractal dimension of steam-cured concrete.

Figure 3 presents the calculated fractal dimensions for various types of steam-cured mortar. The fractal dimension analysis revealed that pore structure complexity increases with higher curing temperatures, directly addressing the research objective of understanding how steam curing affects pore morphology. A rise in fractal dimension at 80 °C indicated a more heterogeneous pore structure, which has detrimental implications for the mortar’s durability. Specifically, the increase in pore irregularity at elevated temperatures was associated with weaker interfacial transition zones and reduced mechanical properties. These findings emphasize the importance of controlling curing temperatures to maintain a less complex, more stable pore structure—an essential factor for ensuring the durability of concrete in real-world applications.

The fractal dimension of the pore structures also varied according to the type of supplementary cementitious material (SCM) used. Mortars containing slag powder exhibited lower fractal dimensions compared to those with fly ash, suggesting a more homogeneous and refined pore network. This implies that the choice of SCM plays a pivotal role in influencing the overall pore complexity and, consequently, the durability of the mortar. By incorporating SCMs such as slag powder, it is possible to achieve a more uniform pore structure, thereby enhancing the mechanical properties and extending the service life of the mortar. Figure 3 illustrates the variations in fractal dimension for different SCM types under various curing conditions, clearly demonstrating how SCM selection impacts pore morphology and long-term performance. These findings carry significant implications for the construction industry, particularly in optimizing the performance of steam-cured concrete. The results underscore the importance of controlling steam curing temperatures to prevent excessive pore irregularity, which negatively impacts mechanical properties and long-term durability. Furthermore, the choice of SCM is critical in refining pore structures and enhancing the overall quality of concrete. The use of SCMs, such as slag powder, can effectively mitigate the negative effects of high-temperature steam curing, leading to more durable and resilient concrete structures. By carefully selecting appropriate SCMs and curing conditions, engineers can improve the performance and longevity of concrete, particularly in precast applications where both strength development and durability are crucial.

### 3.3. Morphology Analysis

The shape of aggregate particles in concrete significantly influences the material’s overall performance. Although steam-cured mortar does not contain traditional aggregates, it possesses a considerable number of distributed pores, the shapes of which directly impact its properties. This section discusses pore morphology through parameters such as shape factor and sphericity. Shape factors include the flatness index and elongation index, both of which are determined by approximating the lengths of a pore’s axes in three perpendicular directions, treated as the axes of an ellipsoid. While these axes are perpendicular, they may not necessarily intersect at a single point [30]. For an ellipsoid with axis lengths L, I, and S, where L > I > S, the flatness index is defined as S/I, and the elongation index is given by I/L. Sphericity (*φ*) is a metric used to quantify how closely the shape of a pore approximates a perfect sphere. It is defined as the ratio of the surface area of a sphere with the same volume as the pore to the actual surface area of the pore. The specific formula for calculating *φ* is provided in [31]:

(4) $$\varphi =\frac{\sqrt[{3}]{\pi {\left(6V\right)}^{2}}}{A}$$

where, *V* is pore volume and *A* is pore surface area.

#### 3.3.1. Shape Factor Analysis

**Figure 4:**
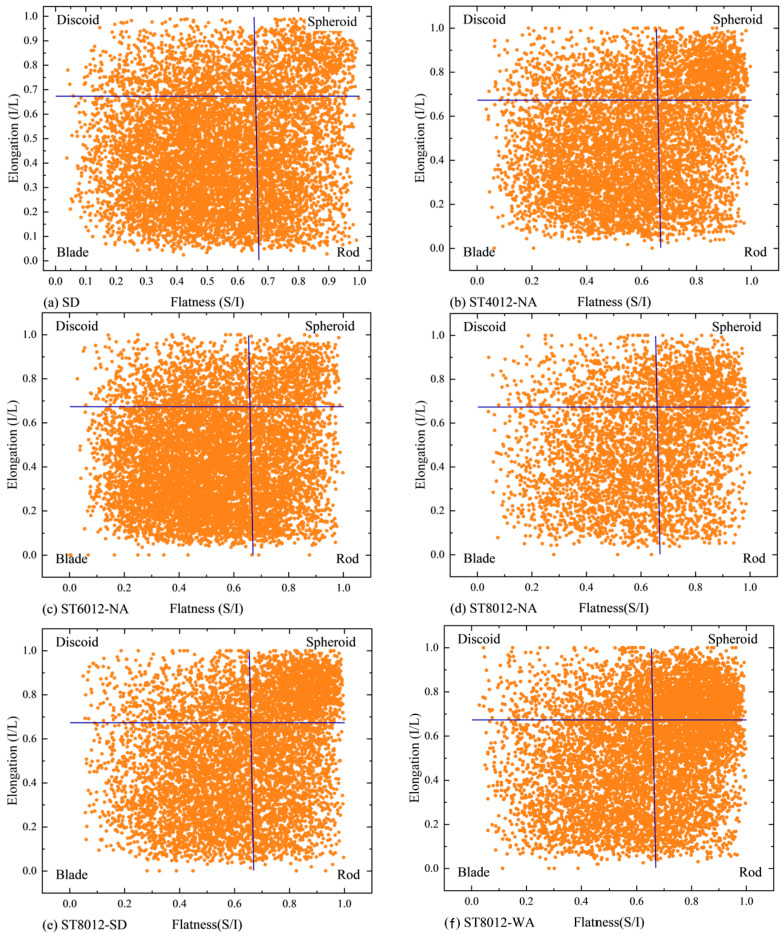
Zingg diagram of six test samples with 0.67 (the blue line) as threshold.

Figure 4 presents a typical Zingg diagram for the mortar samples, classifying the pores based on their flatness and length using a threshold of 0.67. This classification results in four distinct pore shapes: discoid, spheroid, blade, and rod. The specific criteria for this classification are provided in Table 3 [32]. The diagram demonstrates that the pores in the steam-cured samples are distributed across these four shapes, indicating a diverse range of pore morphologies within the samples.

The morphology of the pores was significantly influenced by variations in temperature and curing conditions. For spheroid pores, the proportion initially increased with rising curing temperature, then decreased, and increased again at higher temperatures. This suggests a complex relationship between curing conditions and pore formation. Notably, when compared to natural curing (NA), both standard curing (SD) and water curing (WA) promoted the formation of spheroid pores, with the proportion reaching up to 18% under WA conditions—substantially higher than the approximately 5% observed under NA curing.

**Figure 5:**
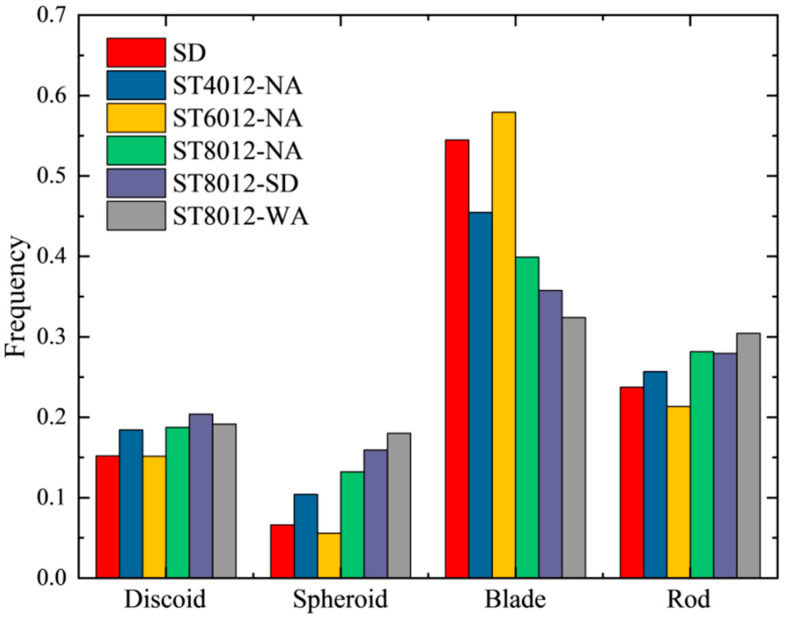
Shape factor distribution of six test samples.

Figure 5 further compares the distribution of the four different pore morphologies across the test samples. The results indicate that spheroid pores constituted the smallest proportion of the total pore volume, ranging from 5% to 18%. The majority of the pores were nonspheroidal, with blade-shaped pores being the most dominant, accounting for approximately 33% to 58% of the total pore volume. This was followed by rod-shaped pores, which comprised 22% to 30% of the total, and discoid pores, which ranged from 15% to 21%. The distribution of discoid and rod pores was relatively uniform, while the disparity between blade and spheroid pores was more pronounced.

The observed variation in pore morphology under different curing conditions highlights the sensitivity of pore structure to thermal treatment. The increased formation of spheroid pores at higher curing temperatures and under water curing suggests that controlling curing conditions can influence the development of more uniform and spherical pore structures, which are associated with improved material performance. The predominance of blade-shaped pores in most samples, particularly at lower curing temperatures, may indicate a less favorable pore structure that could negatively impact the material’s mechanical properties, as irregular pores often correlate with weaker interfacial transition zones. Furthermore, the findings emphasize the importance of postcuring treatments such as water curing, which not only enhances the proportion of spheroid pores but also contributes to improved uniformity in pore structure. This highlights the practical importance of optimizing both the initial steam curing and subsequent postcuring processes to enhance the overall quality and durability of steam-cured concrete. The significant influence of temperature and curing regime on pore morphology suggests that engineers and material scientists should consider these factors when designing concrete mixtures and curing protocols for precast applications [33]. By tailoring curing conditions, particularly the use of water curing after steam curing, it is possible to refine the pore structure and improve the long-term durability and mechanical strength of the material. These findings offer several key implications for the construction industry. First, controlling curing temperatures is crucial for managing pore structure and optimizing the material’s mechanical performance. Second, adopting postcuring practices such as water curing can significantly enhance the uniformity of pore structure and contribute to the long-term durability of concrete. Finally, understanding the relationship between curing conditions and pore morphology can inform the development of more resilient and sustainable concrete materials, particularly in precast concrete production, where rapid strength development and long-term durability are paramount [34]. By applying these insights, practitioners can improve the quality and lifespan of concrete structures, contributing to more efficient and cost-effective construction practices.

#### 3.3.2. Sphericity Analysis

**Figure 6:**
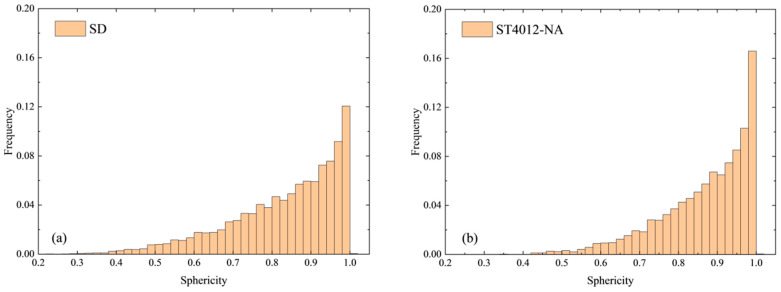

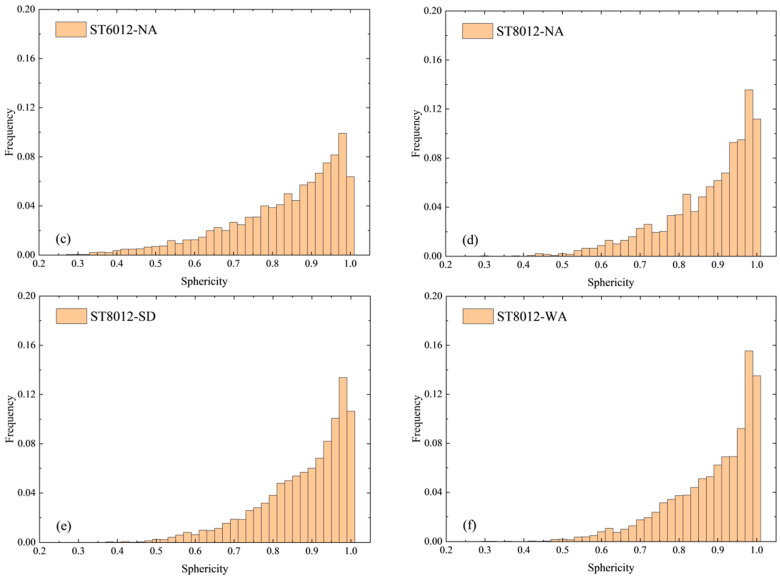
Sphericity distribution of steam-cured mortar samples. (**a**) Sphericity distribution of SD sample. (**b**) Sphericity distribution of ST4012-NA sample. (**c**) Sphericity distribution of ST6012-NA sample. (**d**) Sphericity distribution of ST8012-NA sample. (**e**) Sphericity distribution of ST8012-SD sample. (**f**) Sphericity distribution of ST8012-WA sample.

To gain a deeper understanding of pore morphology in steam-cured mortar, an analysis based on sphericity was conducted. Figure 6 illustrates the distribution of pore sphericity across the steam-cured specimens. The results reveal that pores with a sphericity greater than 0.9 dominated the distribution, accounting for 36% to 55% of the total pores. This suggests that a significant portion of the pores in the mortar specimens approximates a spherical shape.

Temperature and curing conditions were found to have a substantial influence on the distribution of pore sphericity. When compared to standard curing, the proportion of pores with a sphericity greater than 0.9 in the 40 °C sample increased to approximately 47%, indicating that higher curing temperatures promote the transformation of pore shapes toward a more spherical form. However, at 60 °C, the proportion of spherical pores decreased to 37%, suggesting that at this temperature, the pore structure becomes less uniform. As the curing temperature rose further to 80 °C, the proportion of spherical pores increased again, reaching 48%. This rebound highlights the complex interplay between temperature and pore shape evolution.

In addition, improved curing conditions facilitated the transformation of pores toward a more spherical shape, promoting a more uniform pore distribution. Notably, the proportion of near-spherical pores under water curing reached 55%, suggesting that water curing is particularly effective in fostering the evolution of pores into a spherical shape, thus enhancing the overall internal pore structure and uniformity.

**Figure 7:**
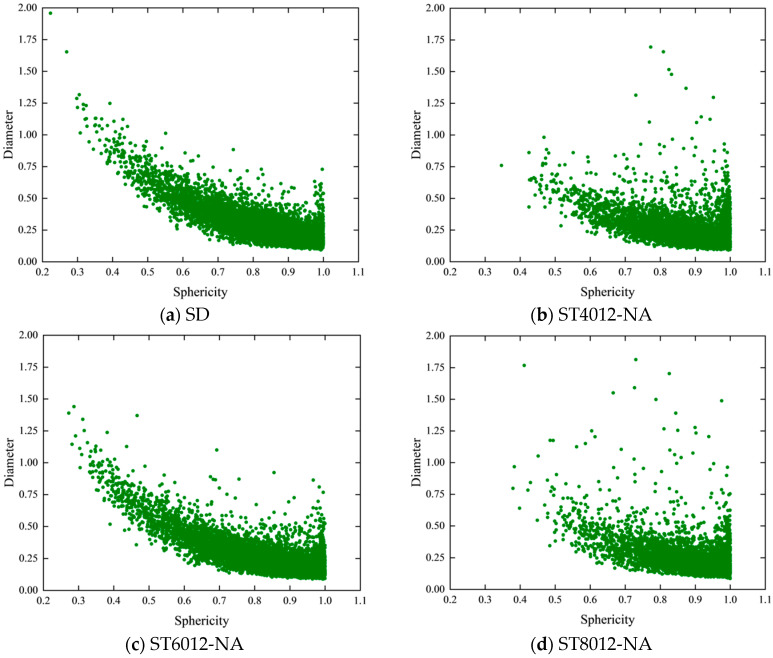

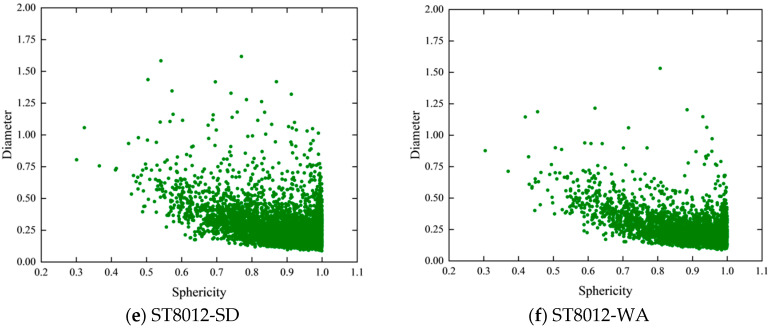
Sphericity-pore diameter scatter diagram of steam-cured concrete.

To investigate whether variations in pore sphericity were associated with pore size, a scatter plot of sphericity versus pore diameter was created, as shown in Figure 7. The plot reveals that smaller diameter pores generally exhibit higher sphericity, regardless of the curing method (steam or standard curing). For samples subjected to standard curing (SD) and steam curing at 60 °C (ST6012-NA), the relationship between diameter and sphericity was relatively consistent, with sphericity increasing as pore diameter decreased. In contrast, specimens subjected to other steam curing conditions exhibited a more variable diameter-sphericity distribution.

Further analysis of pores with a sphericity greater than 0.5 and a diameter greater than 1.0, as shown in Figure 7, reveals that SD specimens contained virtually no pores in this range. In contrast, steam curing resulted in the formation of large-diameter, high-sphericity pores. Specifically, in the ST4012 specimen, 0.5% of the pores fell within this category. As the temperature increased, the presence of large-diameter, high-sphericity pores decreased in the ST6012 specimen, nearly disappearing. However, when the curing temperature was further increased to 80 °C, these pores reappeared, with their proportion rising to 0.58% in the ST8012-NA specimen.

At 80 °C, changes in curing conditions had a notable impact on the occurrence of large-diameter, high-sphericity pores. As the curing conditions improved, the proportion of these pores decreased, with values of 0.4% in ST8012-SD and 0.2% in ST8012-WA. This indicates that these pores are more likely to form under natural curing conditions, further highlighting the importance of controlling the curing process to mitigate the formation of potentially detrimental pore structures. The presence of large-diameter, high-sphericity pores is a distinctive feature of steam-cured specimens.

**Figure 8:**
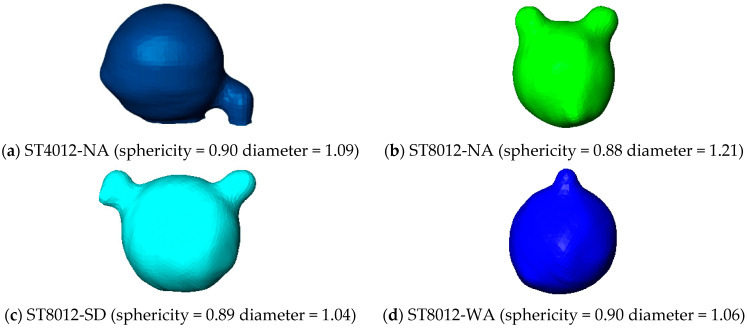
Pore morphology of steam-cured concrete with diameter around 1.0 mm and sphericity around 0.9.

For a more detailed examination of the morphological characteristics of large-diameter, high-sphericity pores formed during steam curing, pores with a diameter of around 1.25 mm and a sphericity of approximately 0.9 were selected for analysis, as shown in Figure 8. The analysis indicates that under steam curing conditions, these pores exhibit a relatively complete spherical shape, with one large-diameter pore connected to one or two smaller pores.

The findings from the sphericity analysis provide valuable insights into how curing conditions, particularly temperature and postcuring methods, influence pore morphology in steam-cured mortar. The increase in spherical pores at higher curing temperatures suggests that optimizing curing conditions can lead to a more uniform pore structure, which is critical for enhancing material performance [35]. The improved distribution of near-spherical pores under water curing emphasizes the importance of postcuring in achieving a more durable and stable pore network. The relationship between pore sphericity and pore size also highlights the significance of controlling pore formation, as smaller pores with higher sphericity contribute to improved mechanical properties. In practical applications, understanding the impact of curing conditions on pore morphology can guide the development of more efficient curing protocols that enhance the mechanical strength and durability of concrete [36].

### 3.4. Correlation Between Pore Characteristics and Mechanical Properties

Porosity is a key characteristic of concrete, with significant variations in porosity, pore size, and pore morphology observed across different curing regimes. Understanding how these variations influence the performance of steam-cured mortar is essential for optimizing its properties. To explore this, the performance of steam-cured mortar was analyzed in terms of pore parameters, specifically focusing on the compressive and flexural strengths at 28 days.

**Figure 9:**
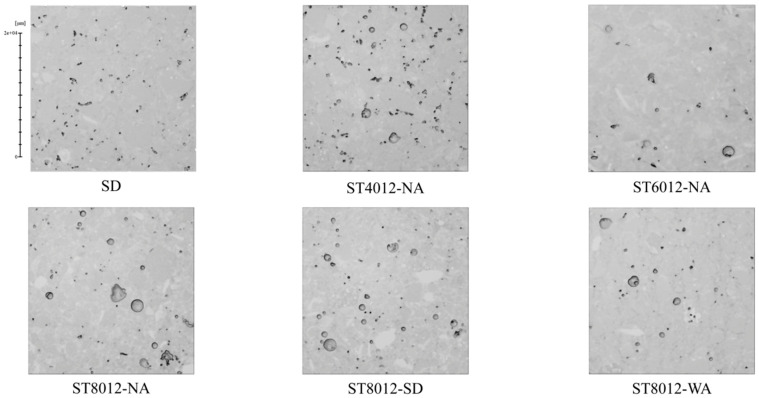
Matrix morphological characteristic of steam-curved mortar.

Figure 9 presents three-dimensional reconstructed images of the mortar matrix under various curing conditions, offering a more intuitive representation of pore distribution within the matrix. Consistent with previous findings, steam-cured mortar exhibited higher porosity, characterized by larger pore volumes and diameters, particularly the presence of large-diameter, high-sphericity pores. However, improved curing conditions contributed to a reduction in internal porosity, resulting in a more cohesive and intact matrix structure.

**Figure 10:**
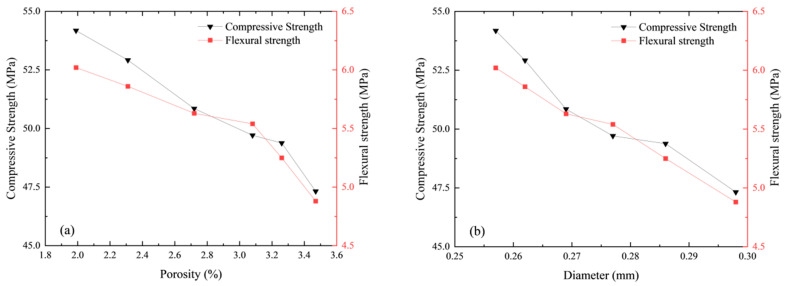
Relationship between pore characters vs. strength. (**a**) porosity vs. strength. (**b**) diameter vs. strength.

To examine the relationship between pore characteristics and the strength of the specimens, both porosity and average pore diameter were calculated, and their correlation with the 28-day compressive and flexural strengths is presented in Figure 10. Figure 10a reveals that the SD specimen exhibited the lowest average porosity at 1.99%, while the ST8012-NA specimen had the highest porosity at 3.47%, with the remaining specimens falling within this range. It was observed that both compressive and flexural strengths decreased as porosity increased, establishing a negative correlation between porosity and strength. The SD specimen achieved the highest compressive strength of 54.18 MPa and flexural strength of 6.02 MPa. In contrast, the steam-cured specimens demonstrated lower strengths, which decreased as the curing temperature increased.

At 80 °C, improved curing conditions helped reduce the average porosity of the specimens, with water curing yielding the highest strengths—showing a 5% increase in compressive strength and a 13.5% increase in flexural strength compared to the natural curing (NA) condition. X-CT analysis revealed that the average pore diameter ranged from 0.257 mm to 0.298 mm. As shown in Figure 10b, the average pore diameter also significantly influenced both compressive and flexural strengths, with strength values decreasing as the average pore diameter increased. Previous analyses have highlighted the presence of large-diameter, high-sphericity pores in steam-cured mortar, which negatively affect its strength. This finding suggests that, in addition to porosity, the average pore diameter plays a critical role in determining the mechanical properties of the material, a conclusion that aligns with similar results found in studies of foamed concrete.

The failure modes of steam-cured specimens were primarily characterized by brittle fracture, with cracks propagating along the interfacial transition zones (ITZs) between the cement matrix and large-diameter pores. As shown in Figure 9, the presence of high-sphericity pores led to localized stress concentrations, accelerating crack initiation. In contrast, specimens subjected to water curing exhibited more uniform crack distribution due to refined pore structures.

These findings underscore the importance of pore structure in determining the mechanical properties of steam-cured mortar. The negative correlation between porosity and compressive strength (*R*² = 0.92, Figure 10a) aligns with the percolation theory, where interconnected pores act as stress concentrators [37]. For instance, the ST8012-NA specimen with the highest porosity (3.47%) exhibited the lowest compressive strength (47.32 MPa), while the SD specimen (porosity 1.99%) achieved 54.18 MPa. The presence of large-diameter, high-sphericity pores further stresses the impact of pore size on the strength of the material, suggesting that strategies aimed at reducing the size and irregularity of these pores can significantly improve the concrete’s mechanical properties. The results also demonstrate the benefits of optimizing curing conditions, especially postcuring treatments like water curing. By reducing porosity and refining the pore structure, these treatments contribute to increased strength and durability, highlighting the practical importance of postcuring in concrete production. Furthermore, understanding the relationship between average pore diameter and strength provides insights into the design of more resilient materials, where pore refinement techniques can be used to enhance both early and long-term performance. These findings have important practical implications for the construction industry, particularly for precast concrete production and the development of high-performance concrete. By integrating these insights into industry practices, engineers can develop more durable and cost-effective concrete solutions for a wide range of applications.

### 3.5. Field Implication

The outcomes of this study have profound implications for the construction industry, particularly in the domain of precast concrete production, where the balance between rapid strength development and long-term durability is critical. By addressing the challenges associated with high-temperature steam curing, the research offers actionable strategies to enhance the structural integrity and service life of concrete components. One key finding is the significant role of postcuring regimes, such as water curing, in mitigating the adverse effects of thermal damage caused by steam curing. The reduction in pore size heterogeneity and the improvement in pore structure uniformity directly translate to enhanced mechanical strength and durability. This insight is particularly relevant for infrastructure projects subjected to aggressive environmental conditions, such as freeze–thaw cycles, chemical attacks, or heavy loading.

The study also underscores the importance of supplementary cementitious materials (SCMs), such as slag and fly ash, in refining pore morphology and reducing thermal stress-induced porosity. By incorporating these materials into concrete mixes, manufacturers can achieve not only superior mechanical performance but also greater sustainability. SCMs reduce reliance on Portland cement, thereby lowering the carbon footprint of construction projects—a critical consideration in modern sustainable engineering practices. In practical terms, the adoption of optimized curing regimes and the strategic use of SCMs offer multiple benefits. For instance, the enhanced pore structure increases resistance to environmental degradation and reduces maintenance requirements, ensuring prolonged service life. Also, the use of SCMs aligns with global efforts to reduce greenhouse gas emissions in the construction industry—meanwhile, reduced maintenance and longer service life of concrete structures lower lifecycle costs for large-scale projects.

This study bridges a critical gap between laboratory research and field applications by providing empirical evidence and practical solutions for mitigating the drawbacks of steam curing. Engineers, manufacturers, and policymakers can leverage these findings to design and implement advanced curing practices that ensure high-performance, durable, and sustainable concrete structures. By optimizing both curing conditions and material compositions, this research lays a robust foundation for innovations in precast concrete technology and promotes resilience in modern infrastructure development.

## 4. Conclusions

This study systematically investigated the effects of steam curing temperatures and postcuring regimes on the pore structure and mechanical properties of high-volume mineral admixture mortar, utilizing advanced X-ray computed tomography (X-CT) for three-dimensional pore characterization. The key findings and implications are summarized as follows:

(1) Elevated steam curing temperatures (e.g., 80 °C) significantly increased porosity (3.47%) and pore size heterogeneity, leading to reduced mechanical strength. For instance, specimens cured at 80 °C under natural conditions (ST8012-NA) exhibited compressive and flexural strengths of 47.32 MPa and 4.88 MPa, respectively. These values are markedly lower than those of standard-cured specimens (SD: 54.18 MPa compressive strength), underscoring the necessity of temperature control below 60 °C to minimize thermal damage.

(2) Water curing (WA) proved highly effective in mitigating the adverse effects of steam curing. Specimens subjected to water curing after 80 °C steam treatment (ST8012-WA) achieved compressive and flexural strengths of 51.72 MPa and 5.85 MPa, representing improvements of 9% and 19.8%, respectively, compared to natural curing. This highlights the critical role of postcuring in refining pore distribution (e.g., reduced pore sphericity variability) and enhancing structural integrity.

(3) The incorporation of slag powder resulted in a more homogeneous pore network (fractal dimension reduced by 12% compared to fly ash-based mixtures), directly improving durability and mechanical performance. Such findings emphasize the importance of SCM selection in optimizing pore morphology, particularly for precast applications requiring both rapid strength gain and long-term resilience.

This study provides actionable strategies for optimizing steam-cured precast concrete, such as controlling curing temperatures below 60 °C and adopting water curing. Future work will focus on (1) long-term durability assessment under carbonation and chloride exposure and (2) integrating machine learning with X-CT data to predict pore structure evolution. These advancements aim to support the development of AI-driven curing systems for sustainable infrastructure.

## Figures and Tables

**Table 1:** Chemical composition of cementitious materials (wt%).

Material	CaO	MgO	SiO_2_	Fe_2_O_3_	P_2_O_5_	Al_2_O_3_	SO_3_
Cement	54.65	2.58	22.07	4.32	1.03	6.30	2.59
Slag	45.09	6.99	27.33	0.45	0.13	13.66	4.03
Fly ash	8.18	0.30	41.11	6.28	1.15	38.62	0.42

**Table 2:** Steam curing conditions and scanning pattern of mortar specimens.

Specimen	Steam Curing Temperature	Steam Curing Age	Scanning System	X-Ray Source Power/W	Voxel Size/mL
ST4012-NA	40 °C	12 h	German Vtomexs micro-focus X-ray CT system	18	38.8
ST6012-NA	60 °C	12 h			
ST8012-NA	80 °C	12 h			
ST8012-WA	80 °C	12 h			
ST8012-SD	80 °C	12 h			

Note: Fine aggregate: 0.1–2 mm. Three specimens were tested for each curing condition.

**Table 3:** Shape factor-based classification of pore size.

Shape	Elongation Index (I/L)	Flatness Index (S/I)	Class
Discoid	>0.67	<0.67	Class I
Spheroid	>0.67	>0.67	Class II
Blade	<0.67	<0.67	Class III
Rod	<0.67	>0.67	Class IV

## Data Availability

The original contributions presented in the study are included in the article, further inquiries can be directed to the corresponding author.

## Abbreviation

2D	Two-dimensional
CT	CT imaging principle computed tomography
MIP	Mercury intrusion porosimetry
NA	Natural curing
SCM	Supplementary cementitious material
SD	Standard curing
SEM	Scanning electron microscopy
ST	Steam curing
ST-NA	Steam curing group—natural curing
ST-SD	Steam curing group—standard curing
ST-WA	Steam curing group—water curing
UHPC	Ultrahigh performance concrete
WA	Water curing
X-CT	X-trray computed tomography
*E_int_*	Internal energy
*E_ext_*	External energy
*E_tot_*	Total energy
*s*	Normalized arc length
*v*	Discrete control point on the active contour curve
*α*	Elasticity coefficient.
*β*	Stiffness coefficient.
*φ*	Sphericity
*V*	Volume
*A*	Area
